# Self-Compassion and Forgiveness Among Individuals in Opioid Substitution Treatment in Greece

**DOI:** 10.1192/j.eurpsy.2025.1601

**Published:** 2025-08-26

**Authors:** E. Kotrotsiou

**Affiliations:** 1 Nursing, Frederick University, Nicosia, Cyprus

## Abstract

**Introduction:**

The work of original research on opioid and heroin depedence is of great interest as it has a serious medical and social impact on society.

Self Compassion and forgiveness are important factors in helping psychosocial support and improving mental health that contributes to the development of person’s character.

**Objectives:**

The purpose of this study is to explore the relationship between self compassion and forgiveness in people who are receiving treatment for opiod depedence.

**Methods:**

An incidental sample of 153 opioid users has been selected for the study.

Data were collected using:

- The Self Compassion Scale.
- the Heartland Forgiveness Scale.

Demographic and social background information was also gathered to complement the primary data.

**Results:**

It has been established that there is a positive relationship between self compassion and the ability to forgive oneshelf or others among people with opiate addiction.

Participants reported appropriate levels of self compassion and forgiveness. Some of the important finding include:

- There is a notable relationship age of first use illicit drugs and the level of forgiveness afforded to others.
- In substance dependency, an inverse relatioship is noted between the level of triazolobenzodiazepine abuse and self forgiveness and self compassion.

**Conclusions:**

There is a chance of improving self kindness and forgiveness even among opioid addicts if these factors may be effective in combating the addictions.

Suggestions for future investigation includes:

- Further evaluation of the age in which a person engages in illicit substances and the level of forgiveness extended to these individuals.

Assessing if the use of benzodiazepine that are not prescribed affects self forgiveness and self compassion.

**Disclosure of Interest:**

None Declared

